# Investigator-Determined Categories for Fever of Unknown Origin (FUO) Compared With International Classification of Diseases–10 Classification of Illness: A Systematic Review and Meta-analysis With a Proposal for Revised FUO Classification

**DOI:** 10.1093/ofid/ofad104

**Published:** 2023-02-24

**Authors:** William F Wright, Jiangxia Wang, Paul G Auwaerter

**Affiliations:** Division of Infectious Diseases, Department of Medicine, Johns Hopkins University School of Medicine, Baltimore, Maryland, USA; Johns Hopkins Biostatistics Center, Johns Hopkins Bloomberg School of Public Health, Baltimore, Maryland, USA; The Sherrilyn and Ken Fisher Center for Environmental Infectious Diseases, Johns Hopkins University School of Medicine, Baltimore, Maryland, USA

**Keywords:** fever, fever of unknown origin, International Classification of Diseases, pyrexia, pyrexia of unknown origin

## Abstract

**Background:**

Classifying fever of unknown origin (FUO) into categorical etiologies (ie, infections, noninfectious inflammatory, oncologic, miscellaneous, and undiagnosed disorders) remains unstandardized and subject to discrepancies. As some disease classifications change, a systematic review of studies would help physicians anticipate the frequency of illness types they may encounter that could influence care.

**Methods:**

We systematically reviewed prospective FUO studies published across the Medline (PubMed), Embase, Scopus, and Web of Science databases from January 1, 1997, to July 31, 2022. We performed a meta-analysis to estimate associated pooled proportions between the investigator-determined choice of disease category and those determined by the International Classification of Diseases, 10th edition (ICD-10), methodology.

**Results:**

The proportion of patients with a difference between the investigator and ICD-10-adjusted noninfectious inflammatory disorder category was 1.2% (95% CI, 0.005–0.021; *P* < .001), and the proportion was similar for the miscellaneous category at 1.5% (95% CI, 0.007–0.025; *P* < .001). The miscellaneous and noninfectious inflammatory disorders categories demonstrated significant across-study heterogeneity in the proportions of patients changing categories, with 52.7% (*P* = .007) and 51.0% (*P* = .010) *I^2^*, respectively.

**Conclusions:**

Adjusting FUO-associated diagnoses by ICD-10 methodology was associated with a statistically significant risk of over- or underestimation of disease category frequency approximation when using a 5 FUO category system. An FUO diagnostic classification system that better reflects mechanistic understanding would assist future research and enhance comparability across heterogenous populations and different geographic regions. We propose an updated FUO classification scheme that streamlines categorizations, aligns with the current understanding of disease mechanisms, and should facilitate empirical decisions, if necessary.

Even though clinicians may commonly think of fever of unknown origin (FUO) diagnoses as falling into 1 of 5 categories (infections, neoplasms, noninfectious inflammatory disorders, miscellaneous conditions, and undiagnosed illnesses), there is no agreement regarding a uniform set of FUO disease classifications [1–3]. In 1907, Cabot classified “long-continued fevers” by statistical methods into 3 main groups: sepsis, tuberculosis, and typhoid [4]. Alt and Baker in 1930 classified patients as either established or unestablished diagnoses [5]. Hamman and Wainwright, in 1936, classified diagnoses as low- or high-grade fever, falling within 5 groups: septicemia, localized septic infections and abscesses, specific infections, diseases of blood-forming organs, or malignant tumors [6].

In their classic 1961 series, Petersdorf and Beeson classified FUO diagnoses as falling within 12 groups: infectious, neoplastic diseases, collagen disease, pulmonary embolization, benign nonspecific pericarditis, sarcoidosis, hypersensitivity states, cranial arteritis, periodic disease, miscellaneous diseases, factitious fever, or no diagnosis [7]. With no criteria for disease subclassification, Durack and Street proposed revisions in 1991, subdividing this problem into 4 distinct FUO types: classic, nosocomial, neutropenic, and HIV-related [8]. In 1997, de Kleijn et al. [9, 10] classified diagnoses as falling within 7 groups: infections, neoplasms, noninfectious inflammatory disorders, drug fever, factitious fever, miscellaneous disorders, or undiagnosed illness.

The observation that disease classification was important for statistical reasons across borders and languages led to the introduction in 1893 of the *International List of Causes of Death* [11]. The work of French physician and botanist Francois Bossier de Sauvages de Lacroix, who in 1763 proposed 10 different illness classifications based on then-current taxonomy botany techniques, led to the first classification based on causes of death. Subsequent investigators modified this method, but the classification system by Jacques Bertillon, Chief of Statistics for Paris, received general approval, with planned published updates once per decade until 1938 [11]. Subsequently, the World Health Organization (WHO) took charge of the classification system in 1948 and renamed it the *International Classification of Diseases* (ICD) for morbidity and mortality [11].

Although a revision cycle was established to keep pace with medical and scientific advancements, versions 1–5 were primarily concerned with mortality causes based on Bertillon's original classification system, which utilized anatomical sites instead of disease etiologies [11]. Subsequent revisions (6–10) shifted to emphasize etiology as the central axis for classification, including causes of mortality and morbidity [11]. The ninth revision, used from 1979 to 1994, accommodated the needs of medical care programs and incorporated a method to classify conditions according to etiology and again by manifestation [11]. Although the order of ICD-10 is similar to ICD-9, it is much more detailed, including nearly 3000 more categories [11]. After several legislative delays, the ICD-10 was adopted in 1995 with enhanced granularity for disease tracking [11]. Countries have adopted ICD updates at various times, and this system has become an integral part of the payment infrastructure of the US health care system [11, 12]. A recent systematic literature review reported that the accuracy of this system was relatively high for capturing the actual diagnosis [13].

In our recent work, we observed discrepancies in the classification of diseases across FUO categories among some recent prospective observational studies [1, 2]. Therefore, we hypothesized that if we adjusted investigator-determined classifications for FUO diagnoses using a standardized methodology such as the ICD-10, this would lead to a lower risk of over- or underestimation of accurate disease category approximation among prospective studies. We propose that this would be especially true for the miscellaneous category, given the diversity of pathophysiology among those diagnoses. The effect will be measured by the proportion of patients who changed category between the 2 versions for each diagnosis. Our ultimate goal with this study was to address the lack of a uniform set of FUO disease classifications, which we identified as a primary unmet need.

## METHODS

This study protocol was registered at the International Prospective Register of Systematic Reviews (PROSPERO; CRD42022308471) [1, 2]. We followed the guidelines of Preferred Reporting Items for Systematic Reviews and Meta-Analyses (Supplementary Table 1: PRISMA Checklist) [14].

### Definitions, Variables, Reclassification of Diseases, and Data Analysis

We defined “investigator-determined classification” as the choice made by the individual who carried out the scientific investigations for FUO research and determined which diagnoses would be grouped under each of the 4 categories (eg, infections, neoplasms, noninfectious inflammatory disorders, or miscellaneous conditions). Investigator-determined classifications for FUO diagnoses were defined as the independent variable. The dependent variable was defined as the true disease category approximation among prospective studies.

**Figure 1.**
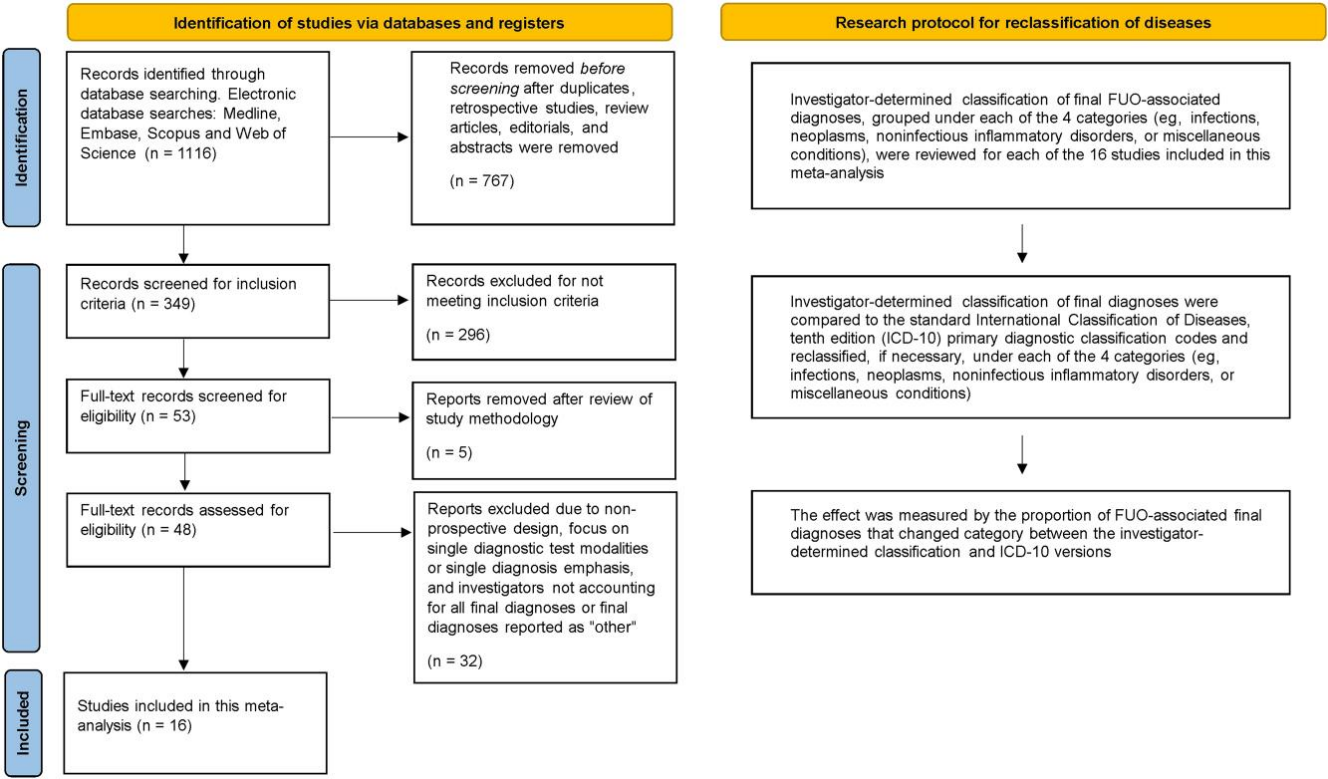
PRISMA flow diagram for searches of databases and schematic of the research protocol for reclassification of diseases.

For this study, investigator-determined diagnoses were reviewed for each study and reclassified, if necessary, based solely on primary etiology codes from the 10th version of the ICD (Figure 1) [11–13]. Both investigator-determined and ICD-10-adjusted diagnoses were then stratified into the following 4 FUO categories: infectious diseases, noninfectious inflammatory disorders, oncology, and miscellaneous conditions [1, 2]. For example, familial Mediterranean fever has an autoinflammatory syndrome primary ICD-10 diagnostic code (M04.1) and would be classified as a noninfectious inflammatory disorder rather than an investigator-determined miscellaneous condition [10, 12]. Data analysis was based upon the proportion of diagnoses that switched categories. We chose the ICD-10 as it was adopted in 1995 and subsequently availabile for WHO member countries, which fit with our study selection protocol [1, 2, 11].

### Study Eligibility Criteria

English- and non-English-language studies were eligible if they were prospective clinical trials, assessed patients meeting any generally accepted FUO definition for adults [3, 7–10], and stratified diagnoses for all categories and etiologic subgroups separately to minimize the chance of unintended selection bias. Additional eligibility information is available in the Supplementary Data.

### Search Strategy

We previously searched Medline (PubMed), Embase, Scopus, and Web of Science from January 1, 1997, to March 31, 2021 [1, 2]. An updated systematic literature search of studies was performed on July 31, 2022. Our search query included terms constructed by a university librarian that had the query strings of (FUO), (fever of unknown origin [Mesh]), (PUO), (pyrexia of unknown origin [Mesh]), (clinical trial), (clinical trial [Publication Type]), and (Prospective Studies [Mesh]).

### Study Selection and Assessment

After titles and abstracts were screened for initial eligibility by W.F.W., full texts of potentially eligible articles were independently reviewed by W.F.W. and P.G.A. Data extraction and quality assessment were performed separately. Any discrepancies were resolved through discussion.

### Data Extraction

Bibliographic information was extracted for the title, author name, year of publication, journal name, FUO inclusion criteria, all primary diagnostic end points by FUO category, secondary end points, setting, and geographical location based upon the 6 WHO regions [1, 2]. Demographics were collected for the number of patients, age, and gender.

### Institutional Review Board Review

This study was exempted from obtaining formal institutional review board approval because it was secondary research of publicly available data.

### Patient Consent

This study does not include factors necessitating patient consent because it was secondary research of publicly available data.

### Statistical Analysis

Extracted data were analyzed with Stata, version 17, using the metaprop command for statistical analysis (Stata Statistical Software: Release 17; StataCorp LLC, College Station, TX, USA). Meta-analysis of investigator-determined and ICD-10-adjusted proportion of diseases contributing to FUO categories was performed using study-specific 95% CIs calculated using the exact method [1, 2]. The pooled proportions across all studies were estimated using the DerSimonian and Laird random-effects model. Freeman-Tukey double arcsine transformation was used to compute the pooled estimate and perform back-transformation on the pooled estimate. Between-study heterogeneity was assessed using the *I^2^* statistic. Heterogeneity was low if *I^2^* was <25.0%, moderate if between 25.0% and 50.0%, and high if >50.0%. This model was also used for subgroup analysis by WHO region. Statistical significance was set at a 2-sided *P*-value of ≤.05.

## RESULTS

### Search Results and Study Characteristics

Of 349 screened articles, 48 publications were included for full-text review (Figure 1). Studies that did not follow a prospective design, concentrated on a single diagnosis and/or diagnostic test modality, did not account for all diagnoses, or had diagnoses listed as “other” by investigators were excluded. Sixteen studies [9, 10, 15–29] were included in our analyses, resulting in 2013 participants. All studies included available data on investigator-determined final diagnoses for each FUO diagnostic category (Supplementary Table 2). Studies were also identified according to WHO geographic region [1, 2]. Supplementary Table 3 summarizes the baseline characteristics of participants from the studies included in our quantitative analyses. Infections (33.5%) and noninfectious inflammatory disorders (22.0%) constituted the most prevalent categories for investigator-determined diagnoses.

### Outcomes of ICD-10-Adjusted Diagnostic Categories

Specific diagnoses requiring adjustment by study and ICD-10 code are listed in Table 1. Analysis of all diagnoses from 5 studies did not require adjustments to new categories [19, 21, 22, 27, 29]. Frequencies of FUO categories after adjustment by ICD-10 coding are listed in Table 2. After adjusting diagnoses, infections (33.7%) and noninfectious inflammatory disorders (23.4%) remained the most prevalent categories.

**Table 1.** Specific Diagnoses Adjusted to the 10th Revision of the International Classification of Diseases (ICD-10)

Study (Country, Region)	Diagnosis	ICD-10 Code	Patients, (n =)	Unadjusted Category	Adjusted Category
Kleijn [9, 10] 1997 (Netherlands, EUR)	Gout/pseudogout	M10.9/M11.2, inflammatory polyarthropathies	3	MISC	NIID
	Mollaret meningitis	G03.2, viral meningitis	2	MISC	INF
	Schnitzler syndrome	D47.2, monoclonal gammopathy	1	NIID	ONC
Kejariwal [15] 2001 (India, SEAR)	Atrial myxoma	D15.1, benign neoplasm of the heart	1	MISC	ONC
	Autoimmune hepatitis	K75.4, other inflammatory liver diseases	1	MISC	NIID
	Granulomatous hepatitis	K75.3, other inflammatory liver diseases	1	MISC	NIID
	Sarcoidosis	D86.9, certain disorders involving the immune mechanism	1	MISC	NIID
Vanderschueren [16] 2003 (Belgium, EUR)	Hemangioma, liver	D18.03, benign neoplasm	1	MISC	ONC
	Schnitzler syndrome	D47.2, monoclonal gammopathy	1	NIID	ONC
	Subacute thyroiditis	E06.1, thyroiditis, inflammation of the thyroid gland	6	MISC	NIID
Saltoglu [17] 2004 (Turkey, EUR)	Granulomatous hepatitis	K75.3, other inflammatory liver diseases	1	MISC	NIID
	Subacute thyroiditis	E06.1, thyroiditis, inflammation of the thyroid gland	1	MISC	NIID
Ergonul [18] 2005 (Turkey, EUR)	Subacute thyroiditis	E06.1, thyroiditis, inflammation of the thyroid gland	3	MISC	NIID
Bleeker [19] 2007 (Netherlands, EUR)	NR	NR	NR	NR	NR
Kucukardali [20] 2008 (Turkey, EUR)	Crohn's disease/ulcerative colitis	K50/K51, noninfective enteritis and colitis	2	MISC	NIID
	Familial Mediterranean fever	M04.1, periodic fever syndromes, autoinflammatory syndromes	2	MISC	NIID
	Gout	M10.9, inflammatory polyarthropathies	1	MISC	NIID
Adil-Khalil [21] 2010 (Iraq, EMR)	NR	NR	NR	NR	NR
Ali-Eldin [22] 2011 (Egypt, EMR)	NR	NR	NR	NR	NR
Mir [23] 2014 (India, SEAR)	Kikuchi disease/necrotizing lymphadenitis	I88.9, nonspecific lymphadenitis, unspecified	2	MISC	NIID
Robine [24] 2014 (France, EUR)	Familial Mediterranean fever	M04.1, periodic fever syndromes, autoinflammatory syndromes	1	MISC	NIID
	Kikuchi disease/necrotizing lymphadenitis	I88.9, nonspecific lymphadenitis, unspecified	1	MISC	NIID
	Rosai-Dorfman disease	D76.3, disorders of blood and blood-forming organs	1	NIID	MISC
	Schnitzler syndrome	D47.2, monoclonal gammopathy	1	NIID	ONC
Naito [25] 2019 (Japan, WPR)	Subacute thyroiditis	E06.1, thyroiditis, inflammation of the thyroid gland	2	MISC	NIID
Rupali [26] 2019 (India, SEAR)	Chronic eosinophilic pneumonia	J82.81, pulmonary eosinophilia, not elsewhere classified	1	NIID	MISC
	Sweet syndrome	L98.2, other disorders of skin and subcutaneous tissue, not elsewhere classified	1	NIID	MISC
Pannu [27] 2021 (India, SEAR)	NR	NR	NR	NR	NR
Cachot [28] 2021 (Spain, EUR)	Gout	M10.9, inflammatory polyarthropathies	1	MISC	NIID
	Subacute thyroiditis	E06.1, thyroiditis, inflammation of the thyroid gland	6	MISC	NIID
Elshalakani [29] 2022 (Egypt, EMR)	NR	NR	NR	NR	NR

Abbreviations: AMR, Region of the Americas; AFR, African Region; EMR, Eastern Mediterranean Region; EUR, European Region; INF, infectious diseases; MIS, miscellaneous causes; NIID, noninfectious inflammatory conditions; NR, no ICD-10 changes required; ONC, oncology/neoplastic conditions; SEAR, Southeast Asian Region; UD, undiagnosed; WPR, Western Pacific Region.

**Table 2.** Outcomes of Prospective Clinical Trials of Fever of Unknown Origin After Adjusting Diagnoses to the 10th Revision of International Classification of Diseases (ICD-10)

Trial Characteristics	Subcategory Characteristics, No. (%)				
Study (Country, Region)	INF	NIID	ONC	MIS	UD
Kleijn [9, 10] 1997 (Netherlands, EUR)	45 (26.9)	42 (25.1)	22 (13.2)	8 (4.8)	50 (29.9)
Kejariwal [15] 2001 (India, SEAR)	53 (53.0)	14 (14.0)	18 (18.0)	1 (1.0)	14 (14.0)
Vanderschueren [16] 2003 (Belgium, EUR)	57 (19.7)	73 (25.1)	31 (10.7)	31 (10.7)	98 (33.8)
Saltoglu [17] 2004 (Turkey, EUR)	51 (58.6)	18 (20.7)	12 (13.8)	0 (0.0)	6 (6.9)
Ergonul [18] 2005 (Turkey, EUR)	42 (52.5)	13 (16.25)	14 (17.5)	2 (2.5)	9 (11.25)
Bleeker [19] 2007 (Netherlands, EUR)	12 (16.0)	16 (22.0)	5 (7.0)	3 (4.0)	37 (51.0)
Kucukardali [20] 2008 (Turkey, EUR)	53 (34.4)	52 (33.8)	22 (14.3)	3 (1.9)	24 (15.6)
Adil-Khalil [21] 2010 (Iraq, EMR)	18 (32.7)	14 (25.4)	9 (16.4)	3 (5.4)	11 (20.0)
Ali-Eldin [22] 2011 (Egypt, EMR)	39 (41.9)	14 (15.1)	28 (30.1)	0 (0.0)	12 (12.9)
Mir [23] 2014 (India, SEAR)	40 (44.0)	13 (14.3)	11 (12.1)	2 (2.2)	25 (27.5)
Robine [24] 2014 (France, EUR)	12 (11.6)	31 (30.1)	4 (3.9)	4 (3.9)	52 (50.5)
Naito [25] 2019 (Japan, WPR)	24 (17.0)	50 (35.5)	22 (15.6)	15 (10.6)	30 (21.3)
Rupali [26] 2019 (India, SEAR)	144 (48.0)	59 (19.7)	64 (21.3)	28 (9.3)	5 (1.7)
Pannu [27] 2021 (India, SEAR)	66 (43.4)	31 (20.4)	33 (21.7)	3 (2.0)	19 (12.5)
Cachot [28] 2021 (Spain, EUR)	15 (17.2)	26 (29.9)	13 (15.0)	7 (8.0)	26 (29.9)
Elshalakani [29] 2022 (Egypt, EMR)	7 (17.5)	6 (15.0)	20 (50.0)	0 (0.0)	7 (17.5)
Total	678 (33.7)	472 (23.4)	328 (16.3)	110 (5.5)	425 (21.1)

Abbreviations: AMR, Region of the Americas; AFR, African Region; EMR, Eastern Mediterranean Region; EUR, European Region; INF, infectious diseases; MIS, miscellaneous causes; NIID, noninfectious inflammatory conditions; ONC, oncology/neoplastic conditions; SEAR, Southeast Asian Region; UD, undiagnosed; WPR, Western Pacific Region.

### Infections

Based on reporting of infections associated with FUO, investigator-determined diagnoses were available for 676 (33.5%) participants. Upon analysis, no diagnosis required reclassification. Among 13 (n = 167 [7.8%]) reported miscellaneous conditions from de Kleijn et al., 2 cases of Mollaret's meningitis were reclassified to infections [9, 10].

### Noninfectious Inflammatory Disorders

For 442 (22.0%) investigator-chosen noninfectious inflammatory disorder diagnoses, 6 of 200 (3.0%) diagnoses from 4 studies were reclassified to another category [7, 8, 16, 24, 26]. Three diagnoses were reclassified to oncology (see the “Cancers” section below). Three diagnoses were reclassified to miscellaneous conditions; Robine et al. [24] reported 1 case of Rosai-Dorfman disease, and another study [26] reported 1 case of chronic eosinophilic pneumonia and 1 case of Sweet syndrome.

### Cancers

Cancers were diagnosed in 322 (16.0%), and no diagnosis required reclassification. Three studies with 139 noninfectious inflammatory diagnoses resulted in 3 cases (2.2%) of Schnitzler syndrome sorted to oncology [9, 10, 16, 24]. Two studies [15, 16] reported 43 total investigator-determined miscellaneous conditions with 1 case each of atrial myxoma and liver hemangioma reclassified to oncology.

### Miscellaneous Disorders

One-hundred forty-eight of 2013 participants (7.4%) were diagnosed with an investigator-chosen miscellaneous condition (range across studies, 0.0%–26.0%). Among 10 studies, 39 of 111 (35.1%) diagnoses were reclassified to another category [9, 10, 15–18, 20, 23–25, 28]. Thirty-five diagnoses (89.7%) were reclassified to noninfectious inflammatory conditions, 2 diagnoses (5.1%) were reclassified as infections (see the “Infection” section above), and 2 diagnoses (5.1%) were reclassified to oncology (see the “Cancer” section above).

Among the miscellaneous diagnoses reclassified to noninfectious inflammatory conditions, 5 studies [16–18, 25, 28] reported 18 cases (n = 76 [23.7%]) of subacute thyroiditis. Three studies [9, 10, 20, 28] reported 5 cases (n = 35 [14.3%]) of gout/pseudogout. Two studies [20, 24] reported 3 cases (n = 13 [23.1%]) of familial Mediterranean fever. One study [20] reported 2 cases (n = 8 [25.0%]) of inflammatory bowel disease. Other diagnoses in 2 studies included 2 cases (n = 7 [28.6%]) of granulomatous hepatitis [15, 17] and 1 each of autoimmune hepatitis [15] and sarcoidosis [15]. Two studies [23, 24] reported 3 cases (n = 9 [33.3%]) of Kikuchi-Fujimoto disease that were reclassified from miscellaneous conditions to noninfectious inflammatory conditions.

### Undiagnosed Diseases

From this study, the overall risk of undiagnosed illness was 21.1% (range, 1.7%–51.0%). As expected, the proportion of undiagnosed illnesses did not differ between investigator-determined and ICD-adjusted diagnoses.

### Pooled Proportions of Patients Changing FUO Disease Categories

**Figure 2.**
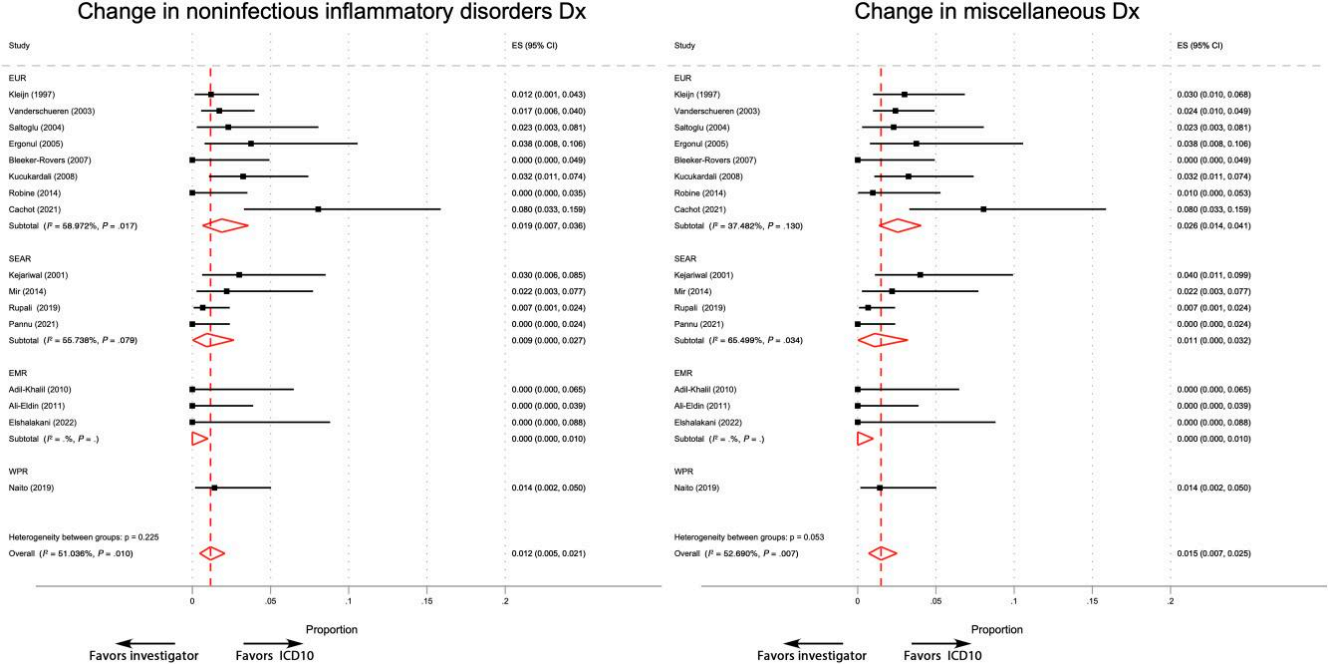
Forest plot graphical display of estimated results from studies addressing FUO noninfectious inflammatory and miscellaneous categorical outcomes adjusted using ICD-10 methodology. Abbreviations: EMR, Eastern Mediterranean Region; ES, effect size; EUR, European Region; FUO, fever of unknown origin; ICD-10, International Classification of Diseases, 10th edition; SEAR, Southeast Asian Region; WPR, Western Pacific Region.

The random-effects pooled proportions with 95% exact CIs and overall pooled estimates for each type of FUO category are shown in Table 3, Figure 2, and Supplementary Figure 1. The difference between investigator-determined and ICD-10-adjusted groups was defined as the proportion of patients who changed category between the 2 versions for each diagnosis.

**Table 3.** Results of Random-Effects Meta-analysis Estimating Pooled Proportions of Patients Changing FUO Disease Categories^a^

Pooled Proportion Comparison of Disease Categories				
	Disease Categories			
	INF	NIID	ONC	MIS
Pooled proportion (95% CI) *P* value for H_0_: proportion = 0	0.000 (0.000–0.001) *P* = 1.00	0.012 (0.005–0.021) *P* < 0.001	0.001 (0.000–0.003) *P* = 0.254	0.015 (0.007–0.025) *P* < 0.001
No. of studies included	16	16	16	16
*I^2^* statistic (95% CI)	0.0% (0.0%–52.3%)	51.0% (13.2%–72.4%)	0.0% (0.0%–52.3%)	52.7% (16.4%–73.2%)
*P* value for heterogeneity among studies	.993	.010	.965	.007
Pooled Proportions by WHO Regions (95% CI), *P* Value for H_0_: Proportion = 0				
EMR	0.000 (0.000–0.010) *P* = 1.00	0.000 (0.000–0.010) *P* = 1.00	0.000 (0.000–0.010) *P* = 1.00	0.000 (0.000–0.010) *P* = 1.00
EUR	0.000 (0.000–0.004) *P* = 0.624	0.019 (0.007–0.036) *P* < 0.001	0.002 (0.000–0.007) *P* = 0.076	0.026 (0.014–0.041) *P* < 0.001
SEAR	0.000 (0.000–0.003) *P* = 1.00	0.009 (0.000–0.027) *P* = 0.027	0.000 (0.000–0.004) *P* = 0.786	0.011 (0.000–0.010) *P* = 0.031
WPR	0.000 (0.000–0.026) *P* = 1.00	0.014 (0.002–0.050) *P* = 0.031	0.000 (0.000–0.026) *P* = 1.00	0.014 (0.002–0.050) *P* = 0.031

Thresholds for interpretation of *I^2^* statistic heterogeneity for this study were low if <25.0%, moderate if between 25.0% and 50.0%, and high if >50.0% [30]. *I^2^* statistic was used to determine the heterogeneity among studies.

Abbreviations: EMR, Eastern Mediterranean Region; EUR, European Region; FUO, fever of unknown origin; INF, infectious diseases; MIS, miscellaneous causes; NIID, non-infectious inflammatory conditions; ONC, oncology/neoplastic conditions; SEAR, Southeast Asian Region; WHO, World Health Organization; WPR, Western Pacific Region.

a Undiagnosed conditions did not change in this study and are not listed.

The noninfectious inflammatory disorders and miscellaneous diagnoses categories demonstrated a small, though statistically different, proportion of patients switching categories. The proportion of patients with a difference between the investigator-determined category and the ICD-10-adjusted noninfectious inflammatory disorder category was 1.2% (95% CI, 0.005–0.021; *P* < .001), and the proportion was similar for the miscellaneous category at 1.5% (95% CI, 0.007–0.025; *P* < .001). The miscellaneous and noninfectious inflammatory disorders categories demonstrated significant across-study heterogeneity in the proportions of patients changing categories, with 52.7% (*P* = .007) and 51.0% (*P* = .010) *I^2^* statistics, respectively. When analyzed by studies performed within WHO geographic regions, only the Eastern Mediterranean region (n = 3 studies) differed compared with other regions.

## DISCUSSION

### Summary of Results

Using the 5 FUO disease category system, we found that ICD-10-adjusted FUO diagnoses, compared with investigator-chosen determination, detected a small but statistically significant risk of over- or underestimating group-level FUO disease category frequency within noninfectious inflammatory disorders and miscellaneous conditions. No significant pooled proportion differences in frequencies between the infection and oncology groups were detected, which speaks to the firmer diagnostic bases of these conditions. Regardless, some disease states may become better understood over time. Decisions to place 1 as a noninfectious inflammatory disorder or in a miscellaneous category could be subject to judgment or controversy.

### Evidence in Context

Discrepancies in classifying diseases within FUO categories were reported in our recent meta-analysis of prospective studies [2]. However, further analyses by FUO diagnostic category or WHO geographic region in that study were not reported. We are unaware of other published studies evaluating frequency differences between investigators and a standardized diagnostic coding system, such as the ICD-10.

Although the primary ICD-10 diagnostic code is based on etiology, the understanding of diseases changes over time. Some disease categories vary in the literature among experts and clinicians, which likely influenced this study's results [2]. For instance, Schnitzler syndrome has a monoclonal gammopathy of undetermined significance (MGUS) primary etiology code. It can progress to Waldenström macroglobulinemia or other lymphoproliferative disorders with a frequency comparable to that of patients with IgM MGUS [31, 32]. Still, it is also very similar to hereditary autoinflammatory diseases such as cryopyrin-associated periodic syndromes (CAPS) [31, 32]. So, whether it should be noninfectious inflammatory or neoplastic is debatable. The primary code for Kikuchi-Fujimoto disease is nonspecific lymphadenitis, but proposed etiologies include either viral or autoimmune mechanisms [33]. For Mollaret's meningitis, the primary etiology code is viral meningitis (eg, varicella zoster, herpes simplex, and West Nile viruses), but Mollaret cells have also been described in other pathologies such as recurrent benign lymphocytic meningitis, sarcoidosis, and Bechet's disease [34]. These controversies in etiology present challenges to sorting FUO diagnoses within the current contemporary categories and might explain the variations observed with the investigator-determined system in our previous study [2].

Given our study results, one must question specifically how certain FUO-associated diseases are classified as miscellaneous or noninfectious inflammatory conditions. Peterdorf's 1961 and 1983 series included miscellaneous conditions such as factitious fever, hematoma, pancreatitis, pulmonary embolism, myelofibrosis, myxoma, and thyroiditis but provided no rationale for placing diagnoses within this category [7, 35]. Additionally, de Kleijn's 1997 series [9, 10] included a miscellaneous disease category but did not provide any fundamental basis for this grouping. More recent studies indicate that proinflammatory cytokines (eg, interleukin 1, 6, and 10) are a central feature to several of these conditions that were classified as miscellaneous conditions (eg, myxoma, pancreatitis, and pulmonary emboli), arguing that they might now better be classified as noninfectious inflammatory conditions [36–38]. Therefore, if an FUO classification system continues to be helpful for both research and direct clinicians’ differential diagnoses, establishing updated, standardized miscellaneous and noninfectious inflammatory group criteria ought to best serve as an optimal classification method. Diseases classified as miscellaneous would then best be reserved for those conditions without an inflammatory component (eg, factious fever), entities for which immunomodulators are not typically employed (eg, drug fever), or disease states that have significant controversies regarding etiology.

### Implications for Clinical Practice With a Proposed New Classification System

While we used a 5–FUO disease category system for this analysis to explore variations, using a different category classification scheme (eg, Petersdorf or de Kleijn) would alter results. However, our choice hews to how clinicians often organize their differential diagnoses of classical FUO [1, 2]. Although this meta-analysis did not support using ICD per se, a new classification of FUO-associated diseases should be warranted to better align long term with ICD classification principles that primarily rely on mechanistic models of physiology [11–13, 39–41]. In other words, ICD works well through its reductionist process function to describe the basics of a disease, its effect on the patient, and perhaps the selection of a therapeutic regimen (eg, mechanism-phenotype-therapy).

However, diagnoses within ICD tend to remain static until there is a significant update. Despite impressively large and longitudinal data sets, this system does not nimbly reflect advancing molecular insights of diseases and lacks requirements for precision medicine, given its rigid hierarchical structure [39–41]. While challenging, a new FUO classification system would also need to include, where available, elements of precision medicine taxonomy recommendations from the National Academy of Sciences (NAS), adapting new science and genomic analyses to clinical medicine and therapeutics [39–41].

FUO-associated disease categories are typically presented as broad classifications for use in an epidemiological context. However, knowing the overall frequency of a category, such as noninflammatory diseases in FUO, will be helpful for patients. For example, FUO patients may lack a discrete noninflammatory diagnosis or end up in a nondiagnostic category. Yet, clinicians may attempt therapeutic trials in such circumstances with drugs such as a corticosteroid. Ample evidence demonstrates that many diseases share relatively common etiologic pathways, particularly autoimmune or autoinflammatory disorders, which ought to better serve as a basis for classification [39–43]. Therefore, we propose a new standardized FUO classification system that better reflects disease mechanisms and will help inform therapeutic decisions (Table 4).

**Table 4.** Proposed Category-Etiology-Treatment (CET) Classification System for Fever of Unknown (FUO) Diagnoses. Examples of FUO-associated diseases are listed in parentheses [1, 2]

I. Infectious Diseases (INF) A. Organism-specified 1. Bacterial (eg, *Mycobacterium tuberculosis*) 2. Fungal (eg, pulmonary zygomycosis) 3. Parasitic (eg, *Plasmodium vivax*) 4. Viral (eg, cytomegalovirus) 5. Other infections (eg, algae) B. Organism-unspecified 1. Antibacterial responsive 2. Antifungal responsive 3. Antiparasitic responsive 4. Antiviral responsive II. Non-infectious Inflammatory Disorders (NIID) A. Autoimmune (eg, Addison's disease, SLE, ANCA vasculitis) B. Autoinflammatory (eg, adult onset Still's disease, Crohn's, FMF, gout, pseudogout, sarcoidosis) C. Mixed autoimmune/autoinflammatory (eg, Behcet's disease) D. Other inflammatory etiology without primary need for immunomodulators or anti-inflammatory agents (eg, chronic pulmonary embolisms) E. Unspecified etiology (eg, Kikuchi disease or response to anti-inflammatory or immunomodulator) III. Non-inflammatory Miscellaneous Disorders (NIMD) A. Behavioral (eg, factitious fever) B. Iatrogenic (eg, drug fever) C. Physiologic (eg, habitual hyperthermia) D. Unspecified etiology IV. Neoplastic Disorders (ONC) A. Benign (eg, atrial myxoma) B. Hematologic origin (eg, lymphoma or leukemia) C. Solid-organ origin (eg, prostatic adenocarcinoma) D. Unspecified etiology (eg, Schnitzler syndrome) V. Undiagnosed FUO syndrome (UFUO)

While a controlled prospective trial would be helpful to validate the proposed revision, we believe this new classification system reflects a synthesis of current knowledge and adapts to the approach of the current ICD classifications. FUO disease-associated conditions will be sorted into the same 5 FUO groups as its main axis (eg, Roman numerals), except that the miscellaneous category has been renamed to exclude noninfectious inflammatory conditions. We believe this modification will reduce the variable assignments used by most researchers and clinicians. Next, diseases will be classified by a common mechanism or primary etiology (eg, alphabetical letters). While these represent the main diagnostic classification methods with this new system, some categories also include treatment response as a subclassification method (eg, category-etiology-therapy system). This especially would help when facing empiric therapeutics for suspected but unconfirmed diseases, such as *Mycobacterium tuberculosis* or undifferentiated inflammatory disorders. Unspecified etiology subclassifications are also included for conditions with significant controversies regarding etiology (eg, Schnitzler syndrome). Finally, with this new system, undiagnosed FUO cases would be reserved exclusively for illnesses without clear evidence of a diagnosis and, if used, lack response to empirical therapeutic trials.

A goal of FUO research should be to improve the accuracy of diagnosis and facilitate testing efficiency. Future research with this new classification system will benefit clinicians as study comparability across heterogenous populations and geographic regions should suffer less variability. The stratification of FUO-associated diseases into improved categorical definitions and subgroups signifies a new FUO taxonomy based on disease mechanisms. It should make research and clinical approaches more realistic.

As it is unclear what classification system is best for FUO diagnoses, lacking a global FUO research network for prospective trials, an alternative approach would employ a consensus panel of FUO experts to reflect and endorse a categorization system. Such an effort is underway. Until such a potential resolution, studies should attempt the mechanistic classification scheme we have proposed that uses our current understanding of mechanisms as a foundation and would guide therapeutics.

### Limitations

Our meta-analysis has limitations. First, the included studies came from different countries and institutions of diverse sizes, contributing to heterogeneity. However, a significant strength of our research is uniformly limiting included studies to prospective cohorts, which is a preferred strategy to strengthen the accuracy of data collection and analyses concerning exposures, potential confounders, end points, and ultimately the accuracy of final diagnoses. Additionally, we performed wide-ranging literature searches on multiple databases. Still, we could have missed studies, which might have increased heterogeneity and reduced accuracy. Finally, the results we present are at the group level, not taken from the patient level. They are subject to variations within particular geographic locations and societies regarding the use of health care resources [2].

## CONCLUSIONS

This study is the first systematic review and meta-analysis to synthesize evidence on how investigator-determined FUO disease classification frequencies may differ compared with a standardized diagnostic classification system. We propose that clinicians and researchers will benefit from a new FUO classification scheme that uses a foundation of mechanistic phenotypes that should help facilitate clinical reasoning and enhance comparability among clinical studies that inform care. Future research is needed to build on these findings and further explore the accuracy of FUO diagnostic coding and categorical classifications to understand if more generalizable conclusions can be drawn.

## Supplementary Material

ofad104_Supplementary_Data

## Supplementary Data

Supplementary materials are available at *Open Forum Infectious Diseases* online. Consisting of data provided by the authors to benefit the reader, the posted materials are not copyedited and are the sole responsibility of the authors, so questions or comments should be addressed to the corresponding author.
