# Supplementary material for: Investigator-Determined Categories for Fever of Unknown Origin (FUO) Compared With International Classification of Diseases–10 Classification of Illness: A Systematic Review and Meta-analysis With a Proposal for Revised FUO Classification
Source: Open Forum Infect Dis. 2023 Feb 24;10(3):ofad104. doi: 10.1093/ofid/ofad104 (PMC10026547; doi:10.1093/ofid/ofad104)
Supplement: ofad104_Supplementary_Data [file ofad104_supplementary_data.docx]

**Supplementary data**

**Supplemental Table 1.** Preferred Reporting Items for Systematic Reviews and Meta-Analyses Checklist

| **Section/topic** | **#** | **Checklist item** | **Reported on page #** |
| --- | --- | --- | --- |
| **TITLE** | | |  |
| Title | 1 | Identify the report as a systematic review, meta-analysis, or both. | Page 1 |
| **ABSTRACT** | | |  |
| Structured summary | 2 | Provide a structured summary including, as applicable: background; objectives; data sources; study eligibility criteria, participants, and interventions; study appraisal and synthesis methods; results; limitations; conclusions and implications of key findings; systematic review registration number. | Page 3-4 |
| **INTRODUCTION** | | |  |
| Rationale | 3 | Describe the rationale for the review in the context of what is already known. | Page 5-7 |
| Objectives | 4 | Provide an explicit statement of questions being addressed with reference to participants, interventions, comparisons, outcomes, and study design (PICOS). | Page 5-7 |
| **METHODS** | | |  |
| Protocol and registration | 5 | Indicate if a review protocol exists, if and where it can be accessed (e.g., Web address), and, if available, provide registration information including registration number. | Page 7-10 |
| Eligibility criteria | 6 | Specify study characteristics (e.g., PICOS, length of follow-up) and report characteristics (e.g., years considered, language, publication status) used as criteria for eligibility, giving rationale. | Page 7-10 |
| Information sources | 7 | Describe all information sources (e.g., databases with dates of coverage, contact with study authors to identify additional studies) in the search and date last searched. | Page 7-10 |
| Search | 8 | Present full electronic search strategy for at least one database, including any limits used, such that it could be repeated. | Page 7-10 |
| Study selection | 9 | State the process for selecting studies (i.e., screening, eligibility, included in systematic review, and, if applicable, included in the meta-analysis). | Page 7-10 |
| Data collection process | 10 | Describe method of data extraction from reports (e.g., piloted forms, independently, in duplicate) and any processes for obtaining and confirming data from investigators. | Page 7-10 |
| Data items | 11 | List and define all variables for which data were sought (e.g., PICOS, funding sources) and any assumptions and simplifications made. | Page 7-10 |
| Risk of bias in individual studies | 12 | Describe methods used for assessing risk of bias of individual studies (including specification of whether this was done at the study or outcome level), and how this information is to be used in any data synthesis. | Page 7-10 |
| Summary measures | 13 | State the principal summary measures (e.g., risk ratio, difference in means). | Page 7-10 |
| Synthesis of results | 14 | Describe the methods of handling data and combining results of studies, if done, including measures of consistency (e.g., I^2^) for each meta-analysis. | Page 7-10 |

**Additional Eligibility Criteria**

Relevant non-English trials were translated with online document translation systems (translate.google.com). Studies were excluded if patients did not fit any accepted FUO definition for adults ^3, 7-10^, were not prospective, did not have full accountability of all diagnoses, or if the risk of bias was judged as unknown. The risk of bias was considered to be unknown if studies did not (1) specify the study type (e.g., prospective or retrospective), (2) provide the FUO criteria used (e.g., consistent with Petersdorf and Beeson ^7^, Durack and Street ^8^, or de Kleijn ^9, 10^ definitions), (3) provide enrollment criteria, (4) specify all outcomes or (5) had substantial amounts of missing data.

**Supplemental Figure 1.** Overall pooled estimates for infections and oncology categories.


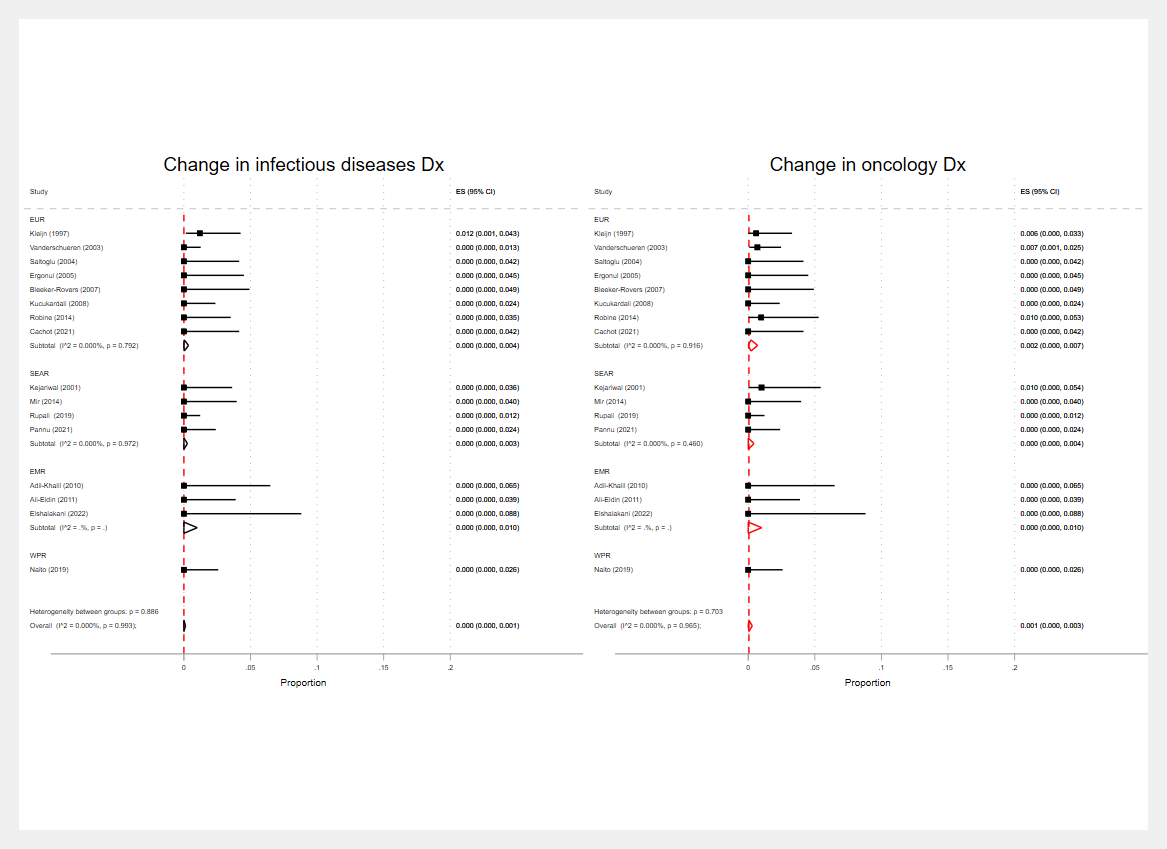


| **Supplemental Table 2. Characteristics of Investigator-Driven Diagnostic Category Outcomes of Analyzed Prospective Clinical Trials of Fever of Unknown Origin** | | | | | | |
| --- | --- | --- | --- | --- | --- | --- |
| **Trial Characteristics** | | **Subcategory* Characteristics, No. (%)** | | | | |
| Study,  (Country, Region§) | Participants  (Female: Male) | INF | NIID | ONC | MIS | UD |
| Kleijn et al, ^9, 10^  1997  (Netherlands, EUR) | 167  (87: 80) | 43  (25.7) | 40  (24.0) | 21  (13.0) | 13  (7.8) | 50  (29.9) |
| Kejariwal et al, ^15^  2001  (India, SEAR) | 100  (41: 59) | 53  (53.0) | 11  (11.0) | 17  (17.0) | 5  (5.0) | 14  (14.0) |
| Vanderschueren et al, ^16^ 2003  (Belgium, EUR) | 290  (126: 164) | 57  (19.7) | 68  (23.4) | 29  (10.0) | 38  (13.1) | 98  (33.8) |
| Saltoglu et al, ^17^  2004  (Turkey, EUR) | 87  (26: 61) | 51  (58.6) | 16  (18.4) | 12  (13.8) | 2  (2.3) | 6  (6.9) |
| Ergonul et al, ^18^  2005  (Turkey, EUR) | 80  (41: 39) | 42  (52.5) | 10  (12.5) | 14  (17.5) | 5  (6.25) | 9  (11.25) |
| Bleeker et al, ^19^  2007  (Netherlands, EUR) | 73  (40: 33) | 12  (16.0) | 16  (22.0) | 5  (7.0) | 3  (4.0) | 37  (51.0) |
| Kucukardali et al, ^20^  2008  (Turkey, EUR) | 154  (71: 83) | 53  (34.4) | 47  (30.5) | 22  (14.3) | 8  (5.2) | 24  (15.6) |
| Adil-Khalil et al, ^21^  2010  (Iraq, EMR) | 55  (28: 27) | 18  (32.7) | 14  (25.4) | 9  (16.4) | 3  (5.4) | 11  (20.0) |
| Ali-Eldin et al, ^22^  2011  (Egypt, EMR) | 93  (48: 45) | 39  (41.9) | 14  (15.1) | 28  (30.1) | 0  (0.0) | 12  (12.9) |
| Mir et al, ^23^  2014  (India, SEAR) | 91  (29: 62) | 40  (44.0) | 11  (12.1) | 11  (12.1) | 4  (4.4) | 25  (27.5) |
| Robine et al, ^24^  2014  (France, EUR) | 103  (49: 54) | 12  (11.6) | 31  (30.1) | 3  (2.9) | 5  (4.9) | 52  (50.5) |
| Naito et al, ^25^  2019  (Japan, WPR) | 141  (78: 63) | 24  (17.0) | 48  (34.0) | 22  (15.6) | 17  (12.1) | 30  (21.3) |
| Rupali et al, ^26^  2019  (India, SEAR) | 300  (106:194) | 144  (48.0) | 61  (20.3) | 64  (21.3) | 26  (8.7) | 5  (1.7) |
| Pannu et al, ^27^  2021  (India, SEAR) | 152  (58: 94) | 66  (43.4) | 30  (19.7) | 32  (21.1) | 5  (3.3) | 19  (12.5) |
| Cachot et al, ^28^  2021  (Spain, EUR) | 87  (41: 46) | 15  (17.2) | 19  (21.8) | 13  (15.0) | 14  (16.1) | 26  (29.9) |
| Elshalakani et al, ^29^  2022  (Egypt, EMR) | 40  (12: 28) | 7  (17.5) | 6  (15.0) | 20  (50.0) | 0  (0.0) | 7  (17.5) |
| Total | 2,013  (881: 1,132) | 676  (33.5) | 442  (22.0) | 322  (16.0) | 148  (7.4) | 425  (21.1) |
| **Abbreviations*: INF, infectious diseases; MIS, miscellaneous causes; NIID, noninfectious inflammatory conditions; ONC, oncology/neoplastic conditions; UD, undiagnosed.  § *Abbreviations*: AMR, Region of the Americas; AFR, African Region; EMR, Eastern Mediterranean Region; EUR, European Region; SEAR, Southeast Asian Region; WPR, Western Pacific Region. [*Ref* 1, 2] | | | | | | |

| **Supplemental Table 3. Demographic Characteristics, Baseline Variables, and Investigator Driven Final Diagnostic Category results for 2,013 Patients with Fever of Unknown Origin** | | |
| --- | --- | --- |
| Characteristic | | Value (n = 2,013) |
| Age, range, y § | | 8-94 |
| Sex, No. (%) | | |
|  | Male | 881 (43.8) |
|  | Female | 1,132 (56.2) |
| No diagnosis, No. (%) | | 425 (21.1) |
| Diagnostic categories, No. (%) | | |
|  | Infectious diseases | 676 (33.5) |
|  | Noninfectious inflammatory diseases | 442 (22.0) |
|  | Cancer | 322 (16.0) |
|  | Miscellaneous | 148 (7.4) |
| § Several reports enrolled patients less than 18 years of age ^9, 10, 15, 17, 20-23, 26, 27, 29^ as long as patients met the adult FUO criteria. | | |
